# Estimating Information Seeking-Behaviour of Public in Malaysia During COVID-19 by Using Google Trends

**DOI:** 10.21315/mjms2020.27.5.16

**Published:** 2020-10-27

**Authors:** Jin Lee Lim, Chong Yau Ong, Beiqi Xie, Lian Leng Low

**Affiliations:** 1 Department of Respiratory Medicine, Hospital Sultanah Aminah Johor Bahru, Johor, Malaysia; 2 SingHealth Community Hospitals, Singapore

**Keywords:** behaviour, coronavirus, Malaysia, internet

## Abstract

The public was reported to be anxious and concerned during the pandemic. It is unknown whether these reactions had a relationship with the statistics of coronavirus disease 2019 (COVID-19) in Malaysia. We used Google Trends (GT) to understand whether the publics’ inquisitiveness towards COVID-19 and its recommended precautionary measures had increased during the initial duration of the pandemic in Malaysia.

## Introduction

Malaysia had not been spared from the coronavirus disease 2019 (COVID-19) pandemic (1). In fact, Malaysia was heavily affected with the highest number of confirmed COVID-19 cases in South East Asia for almost three months until being surpassed by Singapore on 18 April 2020. Malaysia reported its first COVID-19 cases on 25 January, when three China tourists entered Malaysia via Johor from Singapore on 23 January 2020 (2). The public was reported to be anxious and concerned during the pandemic. It is unknown whether these reactions had a relationship with the statistics of COVID-19 in Malaysia. Google Trends (GT) quantifies topics of interest over time in specified geographical location and reports the metrics in relative search volume (RSV) (3). We used GT to understand whether the publics’ inquisitiveness towards COVID-19 and its recommended precautionary measures had increased during the initial duration of the pandemic in Malaysia.

## Methods and Results

**Figure 1:**
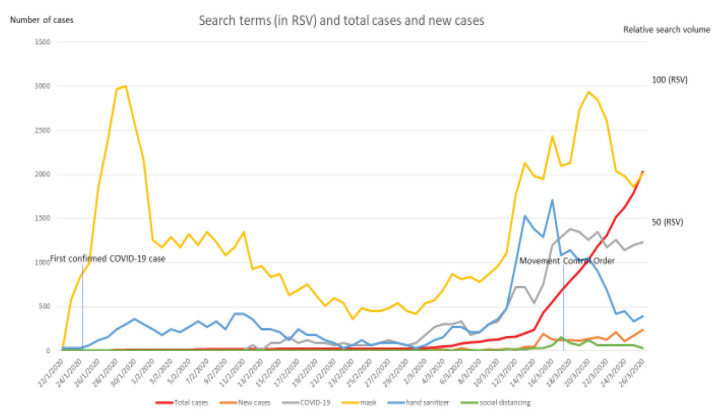
Search terms (in RSV) and the number of reported (new and total) cases

From 22 January 2020 to 26 March 2020, search terms like ‘mask’, ‘hand sanitiser’, ‘social distancing’ and ‘COVID-19’ were retrospectively collected from GT (Google, Palo Alto, CA, USA). Relationship between the precautionary measure and items advocated during the pandemic were measured against the number of new and total cases, as well as total deaths in Malaysia by using the Spearman’s rank correlation coefficient (*r*_s_). Bimodal patterns were observed for mask and hand sanitisers that peaked rapidly following the diagnosis of first COVID-19 case in Malaysia. The second peak corresponded to the third week of March, when the number of new COVID-19 cases rose sharply (Figure 1). The RSV for COVID-19 in GT also showed a peak of search around March 18, leading to March 20.

Mask was found to have garnered more search than COVID-19 itself consistently during the study period. The search COVID-19 showed a very strong correlation with the total number of cases (*r*_s_ = 0.95, *P* = 0) and a significant correlation to the number of new cases diagnosed (*r*_s_ = 0.76, *P* = 0). Social distancing was found to have a strong correlation with the number of cases diagnosed (*r*_s_ = 0.73, *P* = 0) and the total number of deaths (*r*_s_ = 0.79, *P* = 0). Other searches displayed mostly moderate correlation with the temporal statistics in Malaysia (Table 1).

## Discussion

This study showed that the trajectory of COVID-19 numbers in Malaysia roused corresponding searches in Google. The protective consumables of mask and hand sanitiser were the topic of interest for the public. Mask was recognisably the ‘must-have’ item following news on COVID-19 in several affected countries, and the search in Google soared after the confirmation of first COVID-19 case in Malaysia. The interest in hand sanitiser rose subsequently with the awareness of the need for hand hygiene and handwashing. The public was concerned with the adequacy of supply, and the availability of the consumables as the prices of the consumables reached unthinkable rates. The googling activity again increased in the middle of March 2020, when the government announced the Movement Control Order (MCO) starting 18 March 2020. Incidentally, Malaysia received its first COVID-19 mortality on 18 March, spiking the concerns, fears, and anxieties of the public.

Although the RSV for social distancing was pale compared to the consumables, social distancing showed better correlations with the patterns of COVID-19 cases and deaths compared to the search terms mask and hand sanitiser. It was possibly a foreign term to Malaysians until the arrival of COVID-19, and it became more familiar with the public as time went by. The search for public distancing did not increase significantly in the middle of March, likely because of the announcement of MCO in the evening of March 16, 2020. Lockdown and MCO received more than 50,000 and 20,000 searches, respectively, on 17 March, 2020.

Individuals with questions about their health often turn to the internet for information (4). The increased googling activities on the mentioned searches among Malaysians were contemporaneous with the initial COVID-19 statistics in Malaysia. The trend showed that the increase in health-related internet use during the observed period was not unfounded. Similar trend was also observed in Taiwan (5).

We are aware of limitations using GT output to generalise the public’s information seeking behaviour because not all Malaysians have access to the internet. As of 2019, 69% of Malaysians access the internet daily where they read the news, watch a video, and use social media. About 88% of Malaysians were reported to be using smartphones (6).

A better understanding of the Malaysian population’s interest and inquisitiveness to contemporary topics during the pandemic can help the healthcare providers, local authorities, and public to improve content and clarity of education material, as well as addressing the public’s concern through timely dissemination of information and preventive strategies.

## Conclusion

There was a contemporaneous relationship between the search terms related to precautionary and protective measures with the number of cases and deaths of COVID-19 in Malaysia. This opens an opportunity for augmenting education and addressing concerns of the public for future infectious disease pandemics.

## Figures and Tables

**Table 1:** Correlation between search trends with the number of cases and deaths (*r_s_*)

	Sanitiser	Mask	Social distancing	COVID-19
Total cases	0.52801	0.20586	0.67358	0.94848
New cases	0.6913	0.55204	0.73639	0.74663
Total deaths	0.49573	0.48886	0.79392	0.62508
